# Behavior of Human Bone Marrow-Derived Mesenchymal Stem Cells on Various Titanium-Based Coatings

**DOI:** 10.3390/ma9100827

**Published:** 2016-10-12

**Authors:** Chengjuan Qu, Salla Kaitainen, Heikki Kröger, Reijo Lappalainen, Mikko J. Lammi

**Affiliations:** 1 Department of Orthopaedics, Traumatology and Hand Surgery, Kuopio University Hospital, Kuopio 70210, Finland; heikki.kroger@kuh.fi; 2 Department of Integrative Medical Biology, Umeå University, Umeå 90187, Sweden; 3 Department of Applied Physics, University of Eastern Finland, Kuopio 70211, Finland; kaitaine@student.uef.fi (S.K.); reijo.lappalainen@uef.fi (R.L.); 4 Key Laboratory of Trace Elements and Endemic Diseases, National Health and Family Planning Commission, School of Public Health of Health Science Center, Xi’an Jiaotong University, Xi’an 710061, China

**Keywords:** human bone marrow-derived mesenchymal stem cells, ultra-short pulsed laser deposition, surface coating, cell culture, adhesion behavior

## Abstract

The chemical composition and texture of titanium coatings can influence the growth characteristics of the adhered cells. An enhanced proliferation of the human mesenchymal stem cells (hMSCs) would be beneficial. The present study was aimed to investigate whether titanium deposited at different atmospheres would affect the cell growth properties, cellular morphology, and expression of surface markers of hMSCs. Titanium-based coatings were deposited on silicon wafers under oxygen, nitrogen, or argon atmospheres by ultra-short pulsed laser deposition using two different gas pressures followed by heating at 400 °C for 2 h. The characteristics of the coated surfaces were determined via contact angle, zeta potential, and scanning electron microscopy (SEM) techniques. Human MSCs were cultivated on differently coated silicon wafers for 48 h. Subsequently, the cell proliferation rates were analyzed with an MTT assay. The phenotype of hMSCs was checked via immunocytochemical stainings of MSC-associated markers CD73, CD90, and CD105, and the adhesion, spreading, and morphology of hMSCs on coated materials via SEM. The cell proliferation rates of the hMSCs were similar on all coated silicon wafers. The hMSCs retained the MSC phenotype by expressing MSC-associated markers and fibroblast-like morphology with cellular projections. Furthermore, no significant differences could be found in the size of the cells when cultured on all various coated surfaces. In conclusion, despite certain differences in the contact angles and the zeta potentials of various titanium-based coatings, no single coating markedly improved the growth characteristics of hMSCs.

## 1. Introduction

The concept of tissue engineering has made great advances in the field of regenerative medicine, with the idea of using biomaterials and cells to construct new tissues to replace damaged ones in the body. The properties of human mesenchymal stem cells (hMSCs) provide potential for use in regenerative medicine as delivery vehicles for cell-based therapies [1]. They have been considered an alternative cell source for differentiated autologous chondrocytes due to their self-renewal and multipotent capacity to differentiate into different types of cells, including chondrocytes [2,3,4,5,6]. However, the quantity of hMSCs in bone marrow declines dramatically with age. It has been estimated that there is approximately one MSC per 10,000 cells in a newborn child´s bone marrow, whereas a 50-year-old adult has one MSC per 400,000 cells, and an 80-year-old person has one MSC per 2 million cells [7]. Accordingly, it has been shown that the number of colony-forming units harvested per aspirate significantly decreased with age in women [8]. Importantly, the number of hMSCs needed in clinical experiments can be up to 24 million [9]. Thus, it can take a rather long time until enough cells in the monolayer expansion culture for the needs of clinical operations are obtained. Therefore, the enhanced rates of MSC growth in expansion cultures in vitro under environment, which maintain the MSC phenotype and differentiation capacity, would be beneficial for the purposes of tissue engineering.

Silicon has been widely used in biomedical applications [10,11,12,13,14]. It has been shown that the bioactive coating of silicon with bioactive molecules enhanced hMSC proliferation and retained the phenotype of MSCs, as well as the other types of cells [15,16,17,18,19,20]. Silicon substrates were shown to be a feasible novel cell culture material with good biocompatibility properties for myoblast cell adhesion and proliferation [19], and modified silicon has been widely used as a scaffold in cell-based therapy, tissue engineering, or both [15,21,22,23]. Silica nanoparticles have been shown to increase human adipose tissue-derived stem cell proliferation through ERK1/2 activation [24]. Various coatings on silicon wafers have also enhanced the proliferation of hMSCs compared with other types of cells, such as chondrocytes or osteoblasts [16].

Surface properties, such as topography, chemistry, stiffness, roughness, wettability, and energy, have been noted to influence the cell growth and differentiation capacity of stem cells [25,26]. Titanium (Ti) is often used to create artificial joints, pins, and other implants for orthopedic operations, since it does not irritate the human body and is shown to be biocompatible. Titanium dioxide (TiO_2_)-coated CoCrMo has improved the osteogenic differentiation and adhesion of hMSCs [27], and our previous study showed that TiO_2_ coating on cell culture dishes promoted hMSC proliferation without a loss in their chondrogenic differentiation capacity [28]. Cathodic arc plasma-treated Ti has been shown to enhance bone marrow MSC functions [29].

Oxygen and nitrogen are the main gases in the air. The surface coatings on materials are usually exposed to an atmospheric oxygen environment during coating processes or at least during application in cell cultures or as an implant. Ti especially is very reactive and forms at least a thin oxide layer on the surface. Argon is chemically very inactive and has been used to provide an inert atmosphere during deposition. It has been shown that argon protection could effectively reduce the air contaminants on acid-etched Ti implant surfaces and maintain the surface hydrophilicity and biological activity of implants [30,31], enhancing the early bone formation on Ti surfaces [30,32]. Nitrogen is a chemically neutral gas and does not change the biological properties of the samples. Therefore, in the present study, the Ti-based coatings were deposited on silicon or glass in oxygen, nitrogen, or argon atmospheres under two different gas pressures to study how the basic surface properties are affected. Then, the hMSCs were cultivated on these various coatings to investigate whether the different coatings with different surface properties on silicon would affect the proliferation, adhesion, and differentiation of the hMSCs. The main goal was to investigate whether it would be possible to find a coating that would provide the optimal proliferation of hMSCS without a loss in their cellular characteristics. An ultra-short pulsed laser deposition technique was used as a new technology, which allows for the production of well-controlled surface textures in different gas atmospheres.

## 2. Materials and Methods 

### 2.1. Materials

Alpha-modified Eagle’s medium (α-MEM), l-glutamine, fungizone, and penicillin/streptomycin were obtained from Euroclone (Pero, Italy), fetal bovine serum (FBS) from Hyclone (Thermo Scientific, Cramlington, UK), and TrypLE™ from Invitrogen (Carlsbad, CA, USA). Fibroblast growth factor-2 (FGF-2) and transforming growth factor β_3_ (TGF-β_3_) were from Peprotech (London, UK). Toluidine blue was purchased from Serva (Heidelberg, Germany). In coating depositions, high-purity titanium (99.9%) was from Koch-Light Laboratories Ltd. (Colnbrook, UK), and high purity oxygen, nitrogen, and argon were from AGA (HiQ Oxygen 6.0, HiQ Nitrogen 5.0, and HiQ Argon 6.0, Espoo, Finland). The antibodies against CD45 and CD105 were purchased from Development Studies Hybridoma Bank (Iowa City, IA, USA), those against CD73 and CD90 from Abcam (Cambridge, UK), and fluorescein isothiocyanate (FITC)-labeled secondary antibody from Chemicon (Temecula, CA, USA). For immunohistochemical stainings, an Envision+ System-HRP kit (Dako, Glostrup, Denmark) was used for detection. All the other chemicals were from Sigma-Aldrich (St. Louis, MO, USA).

The hMSC culture medium consisted of α-MEM supplemented with 10% FBS, 100 U/mL of penicillin, 100 μg/mL of streptomycin, 2 mM of l-glutamine, 50 µg/mL of 2-phospho-l-ascorbic acid trisodium salt, and 10 ng/mL of FGF-2. The chondrogenic medium contained serum-free α-MEM supplemented with 10 ng/mL of TGF-β_3_ (freshly added), 50 μg/mL of 2-phosphos-l-ascorbic acid trisodium salt, 40 μg/mL of l-proline-HCl, 100 μg/mL of sodium pyruvate, 1% ITS+3 liquid media supplement, and 100 nM of dexamethasone. The osteogenic medium consisted of α-MEM supplemented with 10% FBS, 2 mM of l-glutamine, 100 U/mL of penicillin, 100 μg/mL of streptomycin, 100 nM of dexamethasone, 20 mM of β-glycerophosphate, and 50 μg/mL of 2-phospho-l-ascorbic acid trisodium salt. The adipogenic medium consisted of α-MEM supplemented with 10% FBS, 2 mM of l-glutamine, 100 U/mL of penicillin, 100 μg/mL of streptomycin, 100 nM of dexamethasone, 500 μM of 3-isobutyl-1-methylxanthine, and 100 μM of indomethacin. The control culture medium for the hMSCs was 10% FBS in α-MEM supplemented with 100 U/mL of penicillin and 100 μg/mL of streptomycin. 

### 2.2. Sample Preparation

High purity (100) silicon (Si-Mat, Landsberg am Lech, Germany) and glass microscope slides (Thermo Scientific, Menzel, Braunschweig, Germany) of a size of 76 mm × 26 mm × 0.8 mm were used as substrates. Thin films were deposited using the ultra-short pulsed laser deposition technique (USPLD). A total of six different sets were deposited, and for each set there were coated samples of both silicon and glass. First, the samples were loaded into a vacuum chamber. In vacuum, the sample surfaces were gently cleaned using HiQ Argon (AGA, Espoo, Finland) ion sputtering (SAM-7KV, Minsk, Belarus) before film deposition. For USPLD, we used a Tangerine fs fiber laser (Amplitude Systèmes, Pessac, France). The pulse length was 0.3 ps, with a pulse repetition rate of 2 MHz. Thin films were deposited in oxygen, nitrogen, and argon atmospheres using two different gas pressures—2 × 10^−4^ mbar or 2 × 10^−3^ mbar. As a target, we used high purity titanium. After the depositions, the samples were heated at 400 °C for 2 h in the same atmosphere as that during the deposition. Subsequently, the samples were transitorily ultrasonicated in an ethanol–acetone solution (50:50 in volume).

### 2.3. Contact Angle Measurements

The sessile drop method was used to determine the contact angles of the different surfaces. A custom-made apparatus with a digital camera was used to take a photo of a 10-µL drop of deionized water on each surface. The contact angles were then measured with the GNU image manipulation program (GIMP, version 2.7.3, www.gimp.org). Mean values and standard deviations were then calculated.

### 2.4. Zeta Potential Measurements

The zeta potentials were measured using the electrokinetic analyzer (SurPass, Anton Paar GmbH, Graz, Austria) with the adjustable gap cell. We measured the zeta potentials from the coated and heat-treated silicon samples at a pH of about 7.0, according to the principles of the measurement previously described [28]. The electrokinetic analyzer´s pH meter was used to monitor pH.

### 2.5. Cultivation of Human Mesenchymal Stem Cells (hMSCs)

Human MSCs were isolated from bone marrow materials with permission from the North-Savo Health Care District Ethical Committee (license no. 62/2010), as described previously [28]. Human MSCs were cultured in the MSC culture medium in an incubator at 37 °C with 20% O_2_ tension and 5% CO_2_. When the cells reached 90%–95% confluency in the monolayer culture, they were harvested with trypLE, and 20,000 hMSCs were seeded in the MSC culture medium onto the coated silicon wafers or glass pieces, which were deposited in oxygen, nitrogen, or argon atmospheres from the Ti source. After the cells had been cultivated on various coated materials for 48 h at 37 °C, the samples were collected for MTT and immunocytochemical assays, and for scanning electron microscopic analyses.

### 2.6. Characterization of Human Mesenchymal Stem Cells

To ensure the mesenchymal characterization of the hMSCs from different donors used in this study, the expression of MSC-associated markers of CD73 (1:200), CD90 (1:200), and CD105 (1:200) were examined with an immunocytochemical assay as described in our previous study [33]. The functional characterization of hMSCs was performed using chondrogenic, osteogenic, and adipogenic differentiation assays of hMSCs as previously described [28,33]. 

The chondrogenic differentiation of hMSCs was performed in a pellet culture with 500,000 cells in the chondrogenic medium for 4 weeks. The osteogenic or adipogenic differentiation was carried out in a monolayer culture with 100,000 cells in the osteogenic or adipogenic medium for 4 weeks, respectively. The medium was changed three times per week during the culture period for all differentiation experiments. At the end of the differentiation periods, the cell pellets from chondrogenic differentiation were examined with histological stainings—for proteoglycans (PGs), with toluidine blue staining; for type II collagen, with immunohistochemistry [33,34]. The differentiated cells from osteogenesis and adipogenesis assays were visualized with stainings for alkaline phosphate (ALP) activity and Oil Red O (ORO), respectively [33,34].

### 2.7. Metabolic Activity Measurement

The metabolic activities of the hMSCs cultured on various coated silicon samples were analyzed with an MTT colorimetric assay. After 48-h cultivation, the cells attached to the coated silicon wafers were carefully transferred to a new 24-well plate after washing with phosphate buffered saline (PBS), then 2 mL of a 0.5-mg/mL MTT reagent 3-(4,5-dimethylthiazol-2-yl)-2,5-diphenyltetrazolium bromide was added, and the cells were incubated at 37 °C for 3 h. Finally, the MTT formazan salt was dissolved in 1 mL of dimethylsulphoxide/ethanol (1:1, *v*/*v*), and the absorbances were measured at 595 nm with a 96-well plate reader. Three replicates for every sample were used in the measurement, and the experiment was repeated three times with three different donor cells.

### 2.8. Immunocytochemical Analyses

Immunocytochemical assays on silicon wafers were not successful. Therefore, the characteristic surface antigens of hMSCs were immunostained in cells adhered to the various glass coatings, manufactured in the same conditions as the coatings of silicon wafers. After 48-h cultivation, the cells attached to the coated glass samples were carefully transferred to a new 24-well plate after being washed with PBS; then, the cells were fixed with 4% paraformaldehyde. The fixed cells were further incubated with anti-CD73 (1:200), CD90 (1:200), CD105 (1:200), and CD45 (1:200) antibodies overnight at 4 °C. On the next day, the cells were incubated with a secondary antibody (FITC-labeled goat anti-mouse, 1:200) for 1 h at room temperature in darkness. Finally, the cells were photographed with a fluorescence microscope after incubation with 1 µg/mL of 4′-6-diamidino-2-phenylindone for 15 min at 37 °C [28,33]. The experiment was individually repeated three times with three different donor cells. A 48-h culture time was chosen, since the surfaces were then rather confluent, and longer times were considered to have adverse effects on the cells. This time was thought to be long enough to observe whether loss in the hMSC-specific surface markers would appear.

### 2.9. Scanning Electron Microscopic Analysis of Cell-Free Coated Materials and Cells

Scanning electron microscopic imagings (SEM) and energy-dispersive X-ray spectroscopy (EDS) analysis of the coated surfaces were carried out using a Hitachi S-4800 FE-SEM (Hitachi Science System Ltd., Ibaraki, Japan) equipped with an EDS detector at an accelerating voltage of 5–10 kV.

After 48-h cell cultivation, the cells attached to the coated silicon samples were carefully transferred to a new 24-well plate after washings with PBS; then, the cells were fixed with 2.5% glutaraldehyde in a 0.1-mol/L sodium cacodylate buffer (pH 7.4) for 2 h at room temperature. The samples were further dehydrated with a series of gradually increasing concentrations of ethanol and hexamethyldisilazane. Finally, the samples were covered with gold by sputtering (AGAR auto sputter coater, Agar Scientific, Stansted, UK) for 20 min and monitored with the FE-SEM. The experiment was repeated three times with three different donor cells.

### 2.10. Statistical Analysis

A one-way ANOVA (IBM SPSS Statistics 21, New York, NY, USA) followed by the Bonferroni post-hoc test was used to check the statistical significance of the differences in cell proliferation between the different coated surfaces. The Kruskal–Wallis test was used to examine the statistically significant differences on the contact angle and zeta potential between the different coatings under the same gas pressure or different gas pressures in the same coatings. Significance level *p* < 0.05 was considered statistically significant, and *p* < 0.01 was considered as highly statistically significant.

## 3. Results

### 3.1. Surface Characterization 

The contact angle is used to determine the wettability of a solid surface so that the larger the contact angle (>90°) is, the more hydrophobic the solid surface is. In this study, the contact angles of the coated silicon samples were 90° or higher (Table 1). Significantly increased contact angles of coated silicon were achieved with a nitrogen atmosphere at a lower pressure, and with the presence of argon gas during the deposition (Table 1). The gas pressure had no significant effect on the contact angles of the coated silicon wafers under the oxygen and argon atmospheres at two different gas pressures (Table 1).

The zeta potential, an electrical surface property, depends on the properties of the material surface and the liquid on it. The higher the zeta potential is, the stronger the aggregative stability is, while a lower zeta potential means faster coagulation. In this study, the argon atmosphere at both gas pressures resulted in high negative zeta potential values, as well as coating under lower nitrogen atmospheres (Table 2), while zeta potentials were lowest in samples coated under the oxygen atmosphere (Table 2). The differences in pH values were small during the measurement (Table 2).

**Figure 1:**
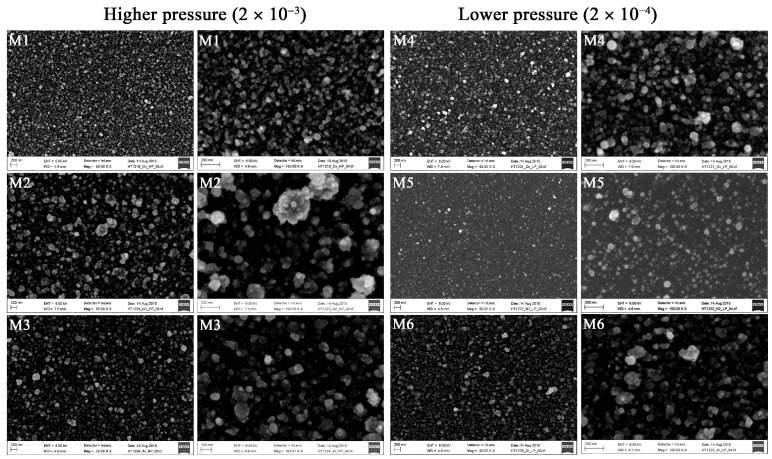
Scanning electron microscopic images of the cell-free surfaces of the titanium-based coatings deposited on silicon. Silicon wafers were coated under higher oxygen (**M1**, scale bar: left 200 nm, right 200 nm), nitrogen (**M2**, scale bar: left 200 nm, right 200 nm), and argon (**M3**, left 200 nm, right 100 nm) pressures and under lower oxygen (**M4**, scale bar: left 200 nm, right 200 nm), nitrogen (**M5**, scale bar: left 200 nm, right 200 nm), and argon (**M6**, scale bar: left 200 nm, right 200 nm) pressures.

Deposition under different conditions affected the surface roughnesses of the coatings on silicon wafers. Surfaces deposited under the higher pressure appeared to have slightly rougher surfaces than those deposited under the lower pressure (Figure 1). The size of the particles in the silicon material deposited in oxygen-plasma under the higher pressure appeared more uniform (Figure 1).

### 3.2. Characterization of the Used hMSCs

**Figure 2:**
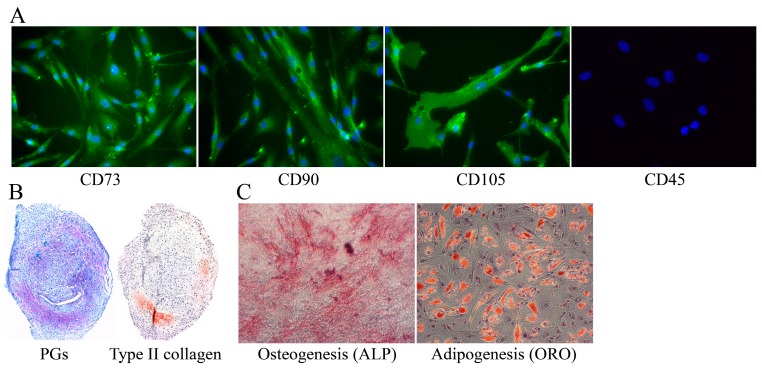
Representative images of characterizations of the human mesenchymal stem cells. (**A**) Immunocytochemical assays show that hMSCs expressed the known MSC-associated markers (CD73, CD90, and CD105), but not leukocyte marker CD45; (**B**) Chondrogenic cell pellets were positively stained with toluidine blue for PGs, and via immunohistochemistry for type II collagen; (**C**) Osteogenic cells in the monolayer culture were stained for alkaline phosphatase activity (ALP), and the adipogenic cells with Oil Red O (ORO) staining. The differentiation assays were performed separately for the cells collected from three different donors.

The hMSCs used in this study were characterized by immunocytochemical stainings of MSC-associated markers—CD73, CD90, and CD105. All three donor hMSCs used in this study expressed surface markers CD73, CD90, and CD105 (Figure 2A), but did not express leukocyte marker CD45 (Figure 2A). The functional characterizations of hMSCs included chondrogenic, osteogenic, and adipogenic differentiation assays. After 4-week chondrogenic differentiation, the cell pellet was stained for PGs and type II collagen (Figure 2B). The osteogenically differentiated cells in the monolayer culture expressed alkaline phosphate activity (ALP) (Figure 2C), and the adipogenic differentiation produced cells that had a high degree of Oil Red O stained fatty droplets (ORO) (Figure 2C).

### 3.3. The hMSC Morphology and Adhesion on Various Coated Silicon Samples

**Figure 3:**
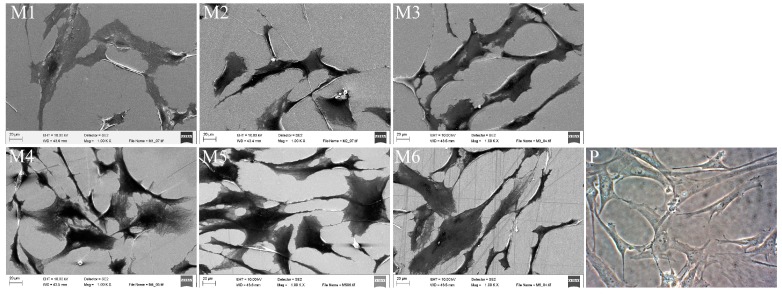
Representative of the morphology of the human mesenchymal stem cells after 48-h cultivation on various coated silicon samples and a polystyrene cell culture plate. Silicon wafers coated under higher oxygen (**M1**), nitrogen (**M2**), and argon (**M3**) pressures and under lower oxygen (**M4**), nitrogen (**M5**), and argon (**M6**) pressures. The scale bar represents 20 μm. (**P**) Shows cells on a polystyrene cell culture plate.

The scanning electron microscopic analysis showed that the hMSCs displayed a fibroblast-like morphology (Figure 3) when the cells were cultivated on all various coated surfaces. The morphology of the hMSCs on various coated surfaces was similar to those cultured as a monolayer culture on standard polystyrene cell culture plates (Figure 3). The hMSCs grown on silicon wafers coated under a nitrogen atmosphere at the higher pressure appeared somewhat smaller in size than the cells grown on other coatings (M2, Figure 3). Therefore, image analysis of the cellular morphology was performed. The data of the cellular area and the perimeter gave some support for the assumption that the hMSCs cultured on the nitrogen-coated surface under high pressure were smallest in size. However, the differences were not statistically significant (Table 3). The circularity of the cells—a value of 1 representing a circular shape—remained almost constant at all coatings, varying in a range between 0.32 and 0.34 (Table 3). This indicates that the cells were mainly spindle-shaped. Solidity describes in geometrical terms the stiffness and deformability of an object. The higher the solidity is, the lower the cell deformability is. In the present study, the solidity values of the hMSCs cultured on various coated silicon samples were between 0.63 and 0.65, and no statistically significant differences could be noticed within various coated silicon samples (Table 3).

### 3.4. The hMSC Proliferation on Various Coated Silicon Samples

**Figure 4:**
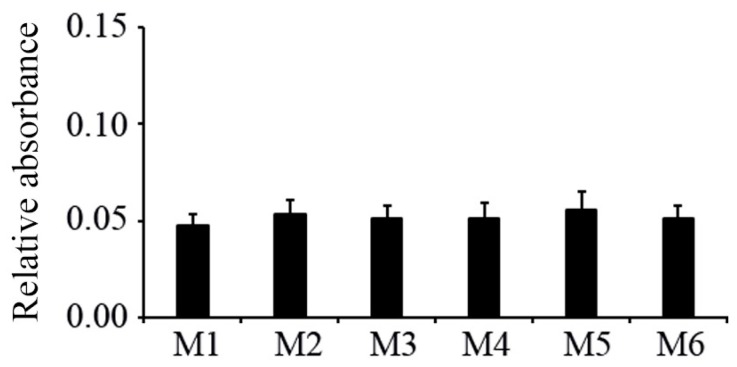
Cell proliferation assayed with MTT after 48-h cultivation of hMSCs on various coated silicon samples (mean ± S.D., *n* = 3). Silicon wafers coated under higher oxygen (M1), nitrogen (M2), and argon (M3) pressures and under lower oxygen (M4), nitrogen (M5), and argon (M6) pressures.

The MTT assay was used to analyze the cell proliferation when the hMSCs were cultivated on various coated silicon samples. The present results from three separate experiments showed no obvious differences in the cell number between any coated silicon samples after cultivation for 48 h (Figure 4).

### 3.5. The Expression of the hMSC-Associated Markers after hMSCs Were Cultivated on Various Coated Silicon Samples

**Figure 5:**
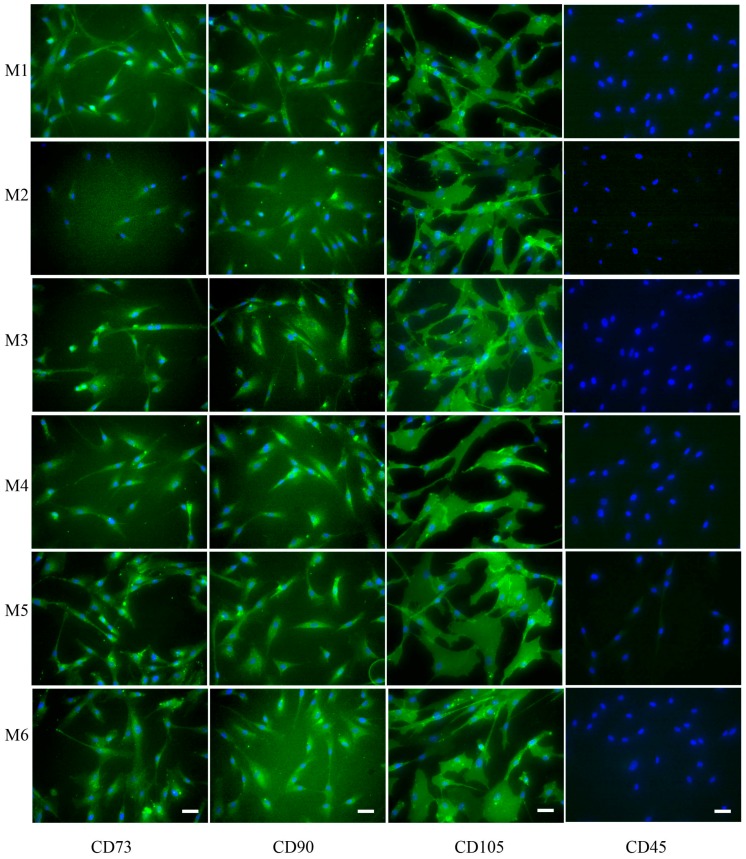
Representative of the immunocytochemical assay of hMSC-associated markers (CD73, CD90, and CD105), and CD45 after the cells were cultivated on various coated glass samples for 48 h. Silicon wafers coated under higher oxygen (M1), nitrogen (M2), and argon (M3) pressures and under lower oxygen (M4), nitrogen (M5), and argon (M6) pressures. Scale bar is 25 µm.

The International Society for Cellular Therapy has stated that the hMSCs must express CD73, CD90, and CD105 and lack expression of CD45, CD34, CD14, or CD19 as the minimal criteria for defining multipotential MSCs [4]. In the present study, the hMSCs from three donors all expressed CD73, CD90, and CD105, but did not express CD45 (Figure 5). However, no noticeable differences could be seen in the expression of CD73, CD90, and CD105 when the cells were cultured on various coated samples (Figure 5).

## 4. Discussion

The hMSCs have gained wide interest in cell-based tissue engineering of bone and articular cartilage, due to their multipotentiality to differentiate into osteoblasts and chondrocytes [28,35]. However, the large number of the chondrogenic hMSCs needed in the clinical application has also been noticed [9]. Our previous study showed that the proliferation rate of the hMSCs significantly increased when they were cultured on TiO_2_-coated cell culture dishes without loss of their capacity for chondrogenic differentiation [28]. Titanium and its alloys have been widely used as implant material in orthopedic application because of their desirable biocompatibility and bioactivity. Moreover, silicon possesses considerable potential in biochemical applications [19,36,37]. Therefore, in the present study, we investigated whether Ti-based coatings deposited in an oxygen, nitrogen, or argon atmosphere on silicon would be beneficial for the proliferation of the hMSCs. High speed ions of laser plasma plume effectively ionize gas atoms. Plasma treatments have been widely used in manufacturing surface modifications, which promote cell adhesion and proliferation [38].

In this study, the coatings with Ti were deposited under three different gas atmospheres, with the idea to investigate whether some coating conditions would yield surfaces optimal for the hMSC proliferation, yet maintain their expression of surface markers typical of hMSCs. The different coating conditions yielded surfaces, which had different characteristics of their contact angle or zeta potential, as well as their roughness. The hMSCs cultured on various Ti-based coatings on silicon wafers showed protrusions and firm adhesion on the coated surfaces. Coatings with Ti under lower nitrogen pressure produced the highest contact angle with relatively smooth surfaces. The cells appeared to be slightly smaller and had a relatively round shape on Ti-based coatings on silicon deposited under higher nitrogen pressure. Thus, the hydrophilicity of the materials obviously facilitates the cell adhesion and spreading [16,39]. Previously, it has been showed that a reduction of 80% in the cell adhesion was apparent when the contact angle was increased from 57° to 122° [40]. A hydrophilic polyurethane matrix promoted chondrogenesis of MSCs [41]. Superhydrophilic vertically aligned carbon nanotubes have permitted the adhesion and maintenance of human chondrocytes [42]. However, the results from our present study indicated that the proliferation of the hMSCs was not significantly different during the 48-h cultivation on various Ti-based coatings on silicon.

It has been shown that the surface roughness affected cell growth, adhesion, spreading, and cell functions [43,44,45,46,47,48,49,50,51,52,53,54,55]. Even though it has been shown that the cell adhesion or proliferation could be enhanced when the cells cultured on rougher surfaces [43,46,49,51,55], the surface roughness could also reduce the cell adhesion, proliferation, or both [47,53,55,56]. Additionally, oxidized Ti samples with rougher surfaces improved the cell adhesion and osteogenic differentiation of the hMSCs [43]. However, no positive effects on the cell proliferation were observed [43]. The proliferation and differentiation of the cells derived from human mandibular bone was enhanced by the surface roughness of the Ti implant [44]. Our present results show that the variations in the surface properties affected by different sample production conditions did not remarkably change the investigated cellular properties, with the exception of a minor difference on the cell adhesion on Ti-based coating deposited in nitrogen under higher pressure compared with the others. This further confirmed our previous studies that the surface roughness did not significantly affect hMSC proliferation [16,26].

Gaseous plasma has been shown to improve biocompatibility by changing the chemical compositions and modifying the surface charge and roughness [57,58]. Oxygen, nitrogen, or argon, a variety of different plasma, can be applied for the surface modification. Oxygen is the most commonly used for plasma treatment of the surface to improve the wettability and controlling of the biocompatibility. It has been shown that the cell proliferation increased by 30% when HEMC-1 cultured on oxygen plasma-treated polymers after 48 h [59]. Oxygen plasma-treated samples could enhance not only the cell adhesion and proliferation, but also the protein adhesion [38]. Nitrogen plasma treatment was more effective than argon and oxygen treatments in the modification of cyclic olefin copolymer microfluidic devices [60]. However, exposure to the air of argon plasma-treated surfaces leads to the incorporation of oxygen or nitrogen species [61,62,63]. Our present study showed that the hMSCs did retain the hMSC phenotype when cultured on Ti-based materials coated in oxygen, argon, or nitrogen, which further confirms our previous study performed with a TiO_2_-coated cell culture dish [28]. 

In conclusion, ultra-short pulsed laser deposition was used as a new technology for Ti-based surface coating deposition under various atmospheres. The present results indicated that the oxygen, nitrogen, or argon atmospheres on Ti-based coatings on silicon wafers produced surfaces, which were different in their surface characteristics but surprisingly appeared to be suitable for the hMSC cultivation and maintenance of their phenotype. Thus, none of the coatings was superior for providing an enhanced proliferation of hMSCs.

## Figures and Tables

**Table 1:** The contact angles of the titanium-based coatings deposited on silicon. Oxygen, nitrogen, and argon gases were used during the depositions under the gas pressures of 2 × 10^−4^ mbar and 2 × 10^−3^ mbar.

Ti-Based Coating on Silicon (Mean ± S.D.)			
Pressure (mbar)	Oxygen	Nitrogen	Argon
2 × 10^−3^ (higher)	93 ± 2	87 ± 3 ^a,b^	115 ± 2
2 × 10^−4^ (lower)	94 ± 2	118 ± 3	110 ± 1

Statistical significances at *p* value < 0.05: ^a^ nitrogen-silicon at higher pressure vs. nitrogen-silicon at lower pressure; ^b^ nitrogen-silicon at higher pressure vs. argon-silicon at higher pressure.

**Table 2:** Zeta potential (ZP) measurement of the coated silicon wafers. Oxygen, nitrogen, and argon gases were used during the depositions under gas pressures of 2 × 10^−4^ mbar and 2 × 10^−3^ mbar.

**Pressure**	Lower Pressure (2 × 10^−4^)			Higher Pressure (2 × 10^−3^)		
**Atmosphere**	Oxygen	Nitrogen	Argon	Oxygen	Nitrogen	Argon
**ZP (mV)**	−30.2 ± 0.3	−35.3 ± 1.6	−41.5 ± 1.9 ^a,c^	−34.5 ± 0.8	−41.6 ± 1.0 ^b,e^	−41.5 ± 2.2 ^d^
**pH**	6.87 ± 0.01	7.01 ± 0.01	7.03 ± 0.04	6.97 ± 0.04	7.02 ± 0.02	7.13 ± 0.04

Statistical significances at *p* value < 0.01: ^a^ argon at lower pressure vs. nitrogen at lower pressure; ^b^ nitrogen at higher pressure vs. nitrogen at lower pressure; ^c^ argon at lower pressure vs. oxygen at lower pressure; ^d^ argon at higher pressure vs. oxygen at higher pressure; ^e^ nitrogen at higher pressure vs. oxygen at higher pressure.

**Table 3:** The cell size and shape-associated parameters (mean ± S.D.) of the human mesenchymal stem cells cultured on various coated silicon samples (*n* = 75). Oxygen, nitrogen, and argon gases were used during the depositions under gas pressures of 2 × 10^−3^ (higher pressure) mbar and 2 × 10^−4^ (lower pressure) mbar.

Materials	Cell Area (µm^2^)	Perimeter (µm)	Circularity	Solidity
M1 (Oxygen, higher pressure)	2079.1 ± 1374.8	293.6 ± 114.2	0.32 ± 0.14	0.65 ± 0.14
M2 (Nitrogen, higher pressure)	2011.2 ± 1331.0	283.2 ± 100.5	0.33 ± 0.16	0.63 ± 0.16
M3 (Argon, higher pressure)	2352.7 ± 1528.1	311.0 ± 133.7	0.33 ± 0.15	0.63 ± 0.17
M4 (Oxygen, lower pressure)	2209.9 ± 1649.5	296.9 ± 112.1	0.32 ± 0.12	0.63 ± 0.14
M5 (Nitrogen, lower pressure)	2371.3 ± 1620.6	297.0 ± 99.0	0.34 ± 0.16	0.64 ± 0.17
M6 (Argon, lower pressure)	2195.3 ± 1349.2	309.8 ± 110.8	0.32 ± 0.17	0.65 ± 0.17
